# Gestações resultantes de violência sexual em meninas de 10-13 anos no Estado do Maranhão, Brasil, no período de 2012-2022: estimativas de frequência, cobertura de notificação e desfechos perinatais

**DOI:** 10.1590/0102-311XPT144325

**Published:** 2026-03-09

**Authors:** Marjory Layla Castro Batista, Rosa Maria Soares Madeira Domingues, Lana dos Santos Meijinhos

**Affiliations:** 1 Escola Nacional de Saúde Pública Sergio Arouca, Fundação Oswaldo Cruz, Rio de Janeiro, Brasil.; 2 Instituto Nacional de Infectologia Evandro Chagas, Fundação Oswaldo Cruz, Rio de Janeiro, Brasil.; 3 Secretaria Municipal de Saúde do Rio de Janeiro, Rio de Janeiro, Brasil.

**Keywords:** Gravidez na Adolescência, Taxa de Fecundidade, Estupro, Notificação de Abuso, Resultado da Gravidez, Pregnancy in Adolescence, Fecundity Rate, Rape, Mandatory Reporting, Pregnancy Outcome, Embarazo en Adolescencia, Índice de Fecundidad, Violación, Notificación Obligatoria, Resultado del Embarazo

## Abstract

O objetivo deste estudo é analisar a taxa de fecundidade, os desfechos da gestação e a cobertura de notificação de estupro em gestações de meninas de 10 a 13 anos no Maranhão, Brasil. Trata-se de estudo ecológico com dados do Sistema de Informações sobre Nascidos Vivos, do Sistema de Informações sobre Mortalidade, do Sistema de Informações Hospitalares do Sistema Único de Saúde e do Sistema de Informação de Agravos de Notificação, referentes ao período de 2012 a 2022, bem como do Censo Demográfico de 2022. Foram analisados os registros de gestações entre meninas com 10 e 13 anos e estimado o número de gestações entre 10 e 13 anos, incluindo gestações com término aos 14 anos, mas com data provável de concepção aos 13 anos. Desfechos em gestações entre 10 e 13 anos foram comparados aos de gestações entre 20 e 29 anos. A cobertura de notificação foi obtida pela comparação entre casos de estupro notificados e o total de gestações registradas e estimadas. Taxa de fecundidade e cobertura de notificação em gestações registradas de 10 a 13 anos foram analisadas segundo Unidade Regional de Saúde (URS). Foi observada taxa de fecundidade de 1,7 por mil, com aumento de 2,5 vezes ao incluir gestações estimadas. Foram notificados 1.410 casos de estupro, correspondendo a uma cobertura de 29,1% das gestações registradas e 11,5% das estimadas. Verificou-se heterogeneidade das taxas de fecundidade e da cobertura de notificação segundo URS de residência. Gestações entre 10 e 13 anos apresentaram até 4 vezes mais desfechos negativos do que gestações entre 20 e 29 anos, mas menor frequência de aborto legal. As desigualdades identificadas e os elevados desfechos negativos em gestações entre 10 e 13 anos reforçam a necessidade de políticas públicas que priorizem a proteção e o atendimento a esse grupo.

## Abstract (en)

The aim of the present study was to analyze the fertility rate, pregnancy outcomes, and rape notification coverage in pregnancies among girls 10 to 13 years of age in the state of Maranhão, Brazil. An ecological study was conducted with data from the Brazilian Information System on Live Births, Brazilian Mortality Information System, Brazilian Hospital Information System, and the Brazilian Information System for Notifiable Diseases in the period from 2012 to 2022 as well as the 2022 Demographic Census. Pregnancies among girls 10 to 13 years of age were analyzed, considering both registered and estimated rates and including those ending at 14 years with a probable date of conception at 13 years of age. Pregnancy outcomes among girls 10 to 13 years of age were compared to those among women 20 to 29 years of age. Notification coverage was determined by comparing reported cases of rape to the total number of registered and estimated pregnancies. The fertility rate and notification coverage in pregnancies among girls 10 to 13 years of age were analyzed according to the Regional Health Unit. A fertility rate of 1.7 per 1,000 was found, with an increase of 2.5 times when including estimated pregnancies. A total of 1,410 cases of rape were reported, corresponding to 29.1% of registered pregnancies and 11.5% of estimated pregnancies. Heterogeneity was found in fertility rates and notification coverage according to the Regional Health Unit of residence. Negative outcomes were up to four times higher in pregnancies involving girls 10 to 13 years of age compared to women 20 to 29 years of age, but the frequency of legal abortion was lower. The inequalities identified and the high negative outcomes in pregnancies involving girls 10 to 13 years of age underscore the need for public policies that prioritize protection and care provided for this group.

## Abstract (es)

El objetivo de este estudio es analizar la tasa de fecundidad, los resultados del embarazo y la cobertura de notificación de violaciones en embarazos entre los 10 y los 13 años en Maranhão, Brazil. Se trata de un estudio ecológico con datos del Sistema de Informacines de Nacidos Vivos, el Sistema de Informaciones de Mortalidad, el Sistema de Información Hospitalaria y el Sistema de Información de Agravios de Notificación, en el período 2012-2022, y el Censo Demográfico de 2022. Se analizaron los embarazos registrados entre los 10 y los 13 años y se estimó el número de embarazos entre los 10 y los 13 años; incluyendo los embarazos que terminaron a los 14 años, pero con una fecha probable de concepción a los 13 años. Se compararon los resultados en embarazos entre los 10 y 13 años de edad con los de embarazos entre los 20 y 29 años de edad. La cobertura de notificación se obtuvo comparando los casos de violación notificados con el total de embarazos registrados y estimados. La tasa de fecundidad y la cobertura de notificación en embarazos registrados de 10 a 13 años se analizaron según la Unidad Regional de Salud (URS). Se observó una tasa de fecundidad de 1,7 por mil, con un aumento de 2,5 veces al incluir los embarazos estimados. Se notificaron 1.410 casos de violación, lo que corresponde a una cobertura del 29,1% de los embarazos registrados y del 11,5% de los estimados. Se observó heterogeneidad en las tasas de fecundidad y en la cobertura de notificación según el URS de residencia. Los embarazos entre los 10 y los 13 años presentaron hasta cuatro veces más resultados negativos que los embarazos entre los 20 y los 29 años, pero una menor frecuencia de abortos legales. Las desigualdades identificadas y los elevados resultados negativos en embarazos entre los 10 y los 13 años refuerzan la necesidad de políticas públicas que prioricen la protección y la atención a este grupo.

## Introdução

O *Código Penal Brasileiro* define as relações sexuais com crianças menores de 14 anos como crime sexual contra vulneráveis, independentemente da capacidade de discernimento da vítima ^1^. Dessa forma, gestações em meninas de 10 a 13 anos no Brasil devem ser consideradas como decorrentes de crime de estupro de vulnerável. Investigar e dar visibilidade a esse tema é uma forma de não permitir que esta ocorrência seja ignorada, postergada e negligenciada, fornecendo dados para que essa violência possa ser evitada.

Além da violação de direitos, a gestação em menores de 14 anos tem maior risco de apresentar desfechos negativos, como aborto, óbito fetal, óbito materno, óbito neonatal, baixo peso ao nascer, prematuridade e transtornos psicológicos e mentais ^2^. Há ainda consequências como a privação da infância, interrupção dos estudos e perpetuação dos ciclos de pobreza ^3^. Estudos anteriores mostram elevada taxa de fecundidade em adolescentes nas regiões Norte e Nordeste do país, estando essa taxa associada à pior condição socioeconômica, iniquidades de renda e de gênero, e menor acesso a serviços de saúde ^4^ ^,^ ^5^ ^,^ ^6^.

O Estado do Maranhão, localizado na Região Nordeste do país, apresenta baixo desenvolvimento socioeconômico, tendo o 26º Índice de Desenvolvimento Humano (IDH) dentre as 27 Unidades Federativas brasileiras. Também apresenta elevada proporção de gestações em adolescentes, baixa cobertura de atenção primária à saúde, baixa cobertura de consultas de pré-natal e elevada incidência de sífilis congênita, revelando situações de vulnerabilidade social e programática ^7^.

Portanto, analisar os casos de gestação em meninas de 10 a 13 anos, que por definição são casos de estupro de vulnerável, por meio dos Sistemas de Informação em Saúde disponíveis, nesse estado do Nordeste, permitirá melhor compreensão desse evento e o fornecimento de dados que futuramente poderão ser utilizados na formulação de estratégias para a prevenção e atenção a essas gestações precoces nas Unidades Regionais de Saúde (URS) do Estado do Maranhão. Sendo os casos de estupro de notificação compulsória, os resultados poderão sensibilizar os profissionais de saúde que realizam o atendimento inicial à vítima sobre a importância de realizar essa notificação, viabilizando o acesso da adolescente a recursos de proteção social e legal.

O objetivo deste estudo é analisar a taxa de fecundidade, os desfechos da gestação e a cobertura de notificação de estupro em gestações de meninas de 10 a 13 anos no Estado do Maranhão, no período de 2012 a 2022.

## Métodos

### Desenho do estudo

Trata-se de estudo ecológico descritivo, tendo como unidade de análise as URS do Estado do Maranhão, utilizando dados disponíveis nos sistemas de informação nacionais para o período 2012-2022. A escolha das URS como unidade de análise foi decorrente de sua utilização para a organização e gestão dos serviços de saúde pela Secretaria Estadual de Saúde do Maranhão. Cada URS agrupa cidades próximas geograficamente e com características econômicas, sociais e demográficas similares, visando uma melhor gestão de saúde.

### Contexto

O Maranhão possui uma população estimada de 6.776.699 habitantes em 2022, sendo o 11º estado mais populoso do país. A população feminina de 10 a 13 anos no Maranhão, de 2012 a 2022, é de 2.851,664, sendo 234.900 no ano de 2022, representando 3,46% da população neste ano ^8^. O estado é composto por 217 municípios, organizados em 19 URS e três macrorregiões de saúde.

### População de estudo

Meninas de 10 a 13 anos, gestantes no período de 2012 a 2022, residentes no Estado do Maranhão.

### Fonte de dados

Foram utilizadas bases não identificadas do Sistema de Informações sobre Mortalidade (SIM), Sistema de Informação sobre Nascidos Vivos (SINASC) e Sistema de Informações Hospitalares do Sistema Único de Saúde (SIH/SUS) e estimativas populacionais a partir do Censo Demográfico realizado pelo Instituto Brasileiro de Geografia e Estatística (IBGE) em 2022. Os dados do SIM, SINASC e SIH/SUS são de acesso público e foram captados pelo pacote *microdatasus* (https://github.com/rfsaldanha/microdatasus) por meio da linguagem de programação estatística R (http://www.r-project.org) ^9^, em 24 de julho de 2024. As estimativas populacionais também são de acesso público, estando disponíveis em http://tabnet.datasus.gov.br/cgi/deftohtm.exe?ibge/cnv/popsvs2024br.def.

Para a identificação dos casos de estupro em meninas de 10 a 13 anos, foram utilizados os dados registrados no SINAN no estado, por ano, no período de 2012 a 2022, por URS de residência. Os dados das notificações estão disponíveis de forma pública em https://datasus.saude.gov.br/transferencia-de-arquivos/ e foram importados e analisados por meio da linguagem de programação estatística R.

Para a comparação dos desfechos gestacionais em meninas de 10 a 13 anos e em mulheres de 20 a 29 anos, foram utilizados dados das gestações de meninas de 10 a 13 anos identificadas nas etapas anteriores e os dados de mulheres de 20 a 29 anos disponíveis no site do Departamento de Informação e Informática do SUS (https://datasus.saude.gov.br/informacoes-de-saude-tabnet/).

### Processamento e análise dos dados em cada etapa do estudo

Para estimar o número de gestações em meninas de 10 a 13 anos por URS do Estado do Maranhão no período de 2012 a 2022, foram considerados todos os possíveis desfechos de uma gestação: nascido vivo, óbito fetal, aborto e óbito materno antes do término da gestação. Em todos os sistemas foram adotados procedimentos para identificar os desfechos de gestação de meninas de 10 a 13 anos no momento do término da gestação, que serão chamados de “casos registrados”.

Seguindo metodologia utilizada anteriormente por Taquette et al. ^2^, foram também identificadas as meninas que tinham 14 anos no momento do término da gestação, mas que teriam 13 anos no momento da concepção. A soma dos casos registrados de 10 a 13 anos e de meninas de 14 anos que engravidaram aos 13 anos será chamada de “casos estimados de gestações de 10 a 13 anos”.

De forma similar ao estudo de Taquette et al. ^2^, no SINASC, foram selecionados os nascidos vivos, com qualquer peso ou idade gestacional, de meninas de 10 a 14 anos residentes no Maranhão, no período 2012-2022. Todos os nascidos vivos de mães de 10 a 13 anos foram considerados como nascidos “registrados”. Para estimar o número de nascidos vivos de mães que tinham 14 anos no momento do parto, cuja concepção ocorreu aos 13 anos, foram realizadas as seguintes etapas:

(a) Exclusão de Declaração de Nascido Vivo (DNV) de mães de 14 anos que não tivessem informações sobre a idade gestacional, data de nascimento ou se a idade calculada a partir da data de nascimento da mãe não correspondesse à idade informada;

(b) Cálculo da idade materna no momento da concepção realizada em duas etapas. Inicialmente, a variável referente às semanas de gestação foi convertida em dias (multiplicada por 7), possibilitando a estimativa da data aproximada da concepção a partir da diferença entre a data de nascimento do recém-nascido e os dias de gestação. A idade materna no momento da concepção foi então calculada pela diferença entre a data estimada da concepção e a data de nascimento da mãe, sendo o resultado convertido em anos completos (divisão da idade em dias por 365).

Dias de gestação = semana gestacional x 7;

Data da concepção = data de nascimento do bebê - dias de gestação;

Idade na concepção = (data da concepção - data de nascimento da mãe) / 365.

O total de “casos estimados de gestações de 10 a 13 anos” foi obtido pela soma das DNV com idade materna menor ou igual a 13 anos e das DNV de mães com 14 anos no momento do parto, cuja idade estimada na concepção era de 13 anos.

No SIM e no SIH/SUS, Taquette et al. ^2^ utilizaram a proporção da população do sexo feminino com idade entre 10 e 13, dentre o total na faixa de 10 a 14 anos do mesmo sexo, para estimar o número de óbitos fetais e de abortos em meninas de 10 a 13 anos dentre o total desses desfechos em meninas de 10 a 14 anos. Óbitos maternos ocorridos antes do término da gestação não foram contabilizados na estimativa do total de gestações em meninas de 10 a 13 anos.

Neste estudo, foram utilizados procedimentos diferentes. No SIM Fetal, foram selecionados todos os óbitos fetais, com qualquer peso ou idade gestacional, de mães que tinham de 10 a 14 anos no momento do término da gestação. Todos os óbitos fetais de meninas de 10 a 13 anos foram considerados “casos registrados”. O SIM Fetal não contém informação sobre a data de nascimento da mãe. Dessa forma, não foi possível calcular a data da concepção, como feito no SINASC. Para estimar o número de óbitos fetais de mães de 14 anos, cuja concepção ocorreu aos 13 anos, consideramos que a mesma proporção observada no SINASC para nascidos vivos ocorreu também para os óbitos fetais.

No SIM Materno, foram selecionados todos os casos de óbito materno ocorridos antes do término da gestação em meninas de 10 a 13 anos. Como a declaração de óbito materno não contém a idade gestacional no momento do óbito, não foi possível estimar a data da concepção e consequentemente o número de meninas que faleceram aos 14 anos, mas que engravidaram aos 13 anos.

No SIH/SUS, foram identificadas as internações com diagnóstico principal de abortamento (Classificação Internacional de Doenças, 10ª revisão - CID-10, códigos O00 a O08) em meninas de 10 a 14 anos. Todas as internações por aborto em meninas de 10 a 13 anos foram considerados casos registrados. Como a Autorização da Internação Hospitalar não contém informações sobre a data do abortamento nem sobre a idade gestacional no término da gestação, utilizamos os seguintes procedimentos para estimar internações por aborto de meninas de 14 anos que teriam engravidado aos 13 anos:

(a) Consideramos a data da internação como data do término da gestação;

(b) Consideramos duas idades gestacionais: 6 semanas (limite mínimo para identificação do saco gestacional por ultrassonografia obstétrica) e 21 semanas (limite máximo para classificação do desfecho da gestação com uma perda fetal precoce).

Da mesma forma, como no SINASC, subtraímos a idade gestacional em dias (semana gestacional x 7) da data da internação e, posteriormente, a data de nascimento da mãe da data estimada da concepção dividida por 365 dias. Meninas de 14 anos com data de concepção estimada aos 13 anos foram incluídas no total de gestações de 10 a 13 anos estimadas.

Nos três sistemas (SINASC, SIM e SIH/SUS), as gestações registradas em meninas de 10 a 13 anos foram selecionadas por município de residência e agrupadas nas suas respectivas URS. O total de gestações registradas considerou a soma de nascidos vivos, óbitos fetais, óbitos maternos antes do término da gestação e internações por abortamento.

Para o estado, foram gerados dois totais do número de gestações: um contendo os “casos registrados” (meninas de 10 a 13 anos no momento do término da gestação) e outro os “casos estimados” (meninas de 10 a 13 anos no momento da gestação + meninas de 14 anos com data da concepção estimada aos 13 anos).

Para cálculo da taxa de fecundidade específica de meninas de 10 a 13 anos por URS e para o estado, foi utilizada como numerador a média do número de gestações registradas em meninas de 10 a 13 anos, no período 2012-2022, em cada URS e no estado, e como denominador, a população estimada de meninas de 10 a 13 anos por URS e no estado, no período 2012-2022. Para o cálculo da taxa de fecundidade estimada em meninas de 10 a 13 anos para o Estado do Maranhão, utilizamos como numerador a média do número de gestações estimadas no estado, e como denominador, a média da população de meninas de 10 a 13 anos residentes no estado no período de 2012 a 2022.

Para estimar a cobertura de notificação de casos de estupro em meninas de 10 a 13 anos por URS no período 2012-2022, utilizamos no numerador os casos de notificações de estupro em meninas de 10 a 13 anos identificados no SINAN por URS no período, e no denominador, o número de gestações registradas em meninas de 10 a 13 anos por URS no período. Para identificação das notificações de casos de estupro em meninas de 10 a 13 anos, foram selecionadas, dentre as notificações de violência sexual, aquelas em indivíduos do sexo feminino, na faixa etária de 10 a 13 anos, residentes no Estado do Maranhão, no período 2012-2022, com preenchimento das variáveis “SEX_ESTUPRO (1=sim)”. As notificações foram selecionadas segundo município de residência da mulher e posteriormente agrupadas segundo URS, sendo considerada a data de ocorrência da agressão que consta na notificação. Para a cobertura de notificação no estado, foram realizados dois cálculos, um utilizando no denominador apenas o total de casos de “gestações registradas” e no outro o total de “gestações estimadas” em meninas de 10 a 13 anos.

Foram analisados os seguintes desfechos das gestações registradas em meninas de 10 a 13 anos e mulheres de 20 a 29 anos no Estado do Maranhão no período 2012-2022:

Proporção de baixo peso ao nascer: número de nascidos vivos com peso < 2.500g em relação ao total de nascidos vivos;

Proporção de prematuridade: número de nascidos vivos com idade gestacional < 37 semanas em relação ao total de nascidos vivos;

Razão “aborto legal/notificação de estupro”: razão entre o número de gestações que resultaram em aborto legal (número de internações hospitalares no SIH/SUS contendo CID-10 O04) e o número total de casos notificados de estupro;

Razão de mortalidade materna (RMM): número de óbitos maternos dividido pelo total de nascidos vivos e multiplicado por 100.000;

Taxa de mortalidade fetal: número de óbitos fetais dividido pela soma de óbitos fetais e nascidos vivos e multiplicado por 1.000;

Taxa de mortalidade neonatal total: número de óbitos neonatais (0 a 27 dias) dividido pelo número de nascidos vivos e multiplicado por 1.000.

Os valores encontrados foram comparados aos observados em mulheres de 20 a 29 anos, faixa etária de menor risco para desfechos perinatais negativos como baixo peso ao nascer e mortalidade ^10^.

### Aspectos éticos

O projeto foi submetido ao Comitê de Ética em Pesquisa da Escola Nacional de Saúde Pública Sergio Arouca, Fundação Oswaldo Cruz (ENSP/FIOCRUZ), em 11 de julho de 2024, e recebeu o parecer de dispensa de apreciação ética nº 11/2024, em 24 de julho de 2024. A pesquisa foi realizada com dados e informações de bases secundárias de domínio público, estando dispensada de apreciação ética pelo Sistema CEP-CONEP, conforme o Art. 1º, parágrafo único, item III da *Resolução nº 510/2016* do Conselho Nacional de Saúde.

## Resultados

**Figura 1:**
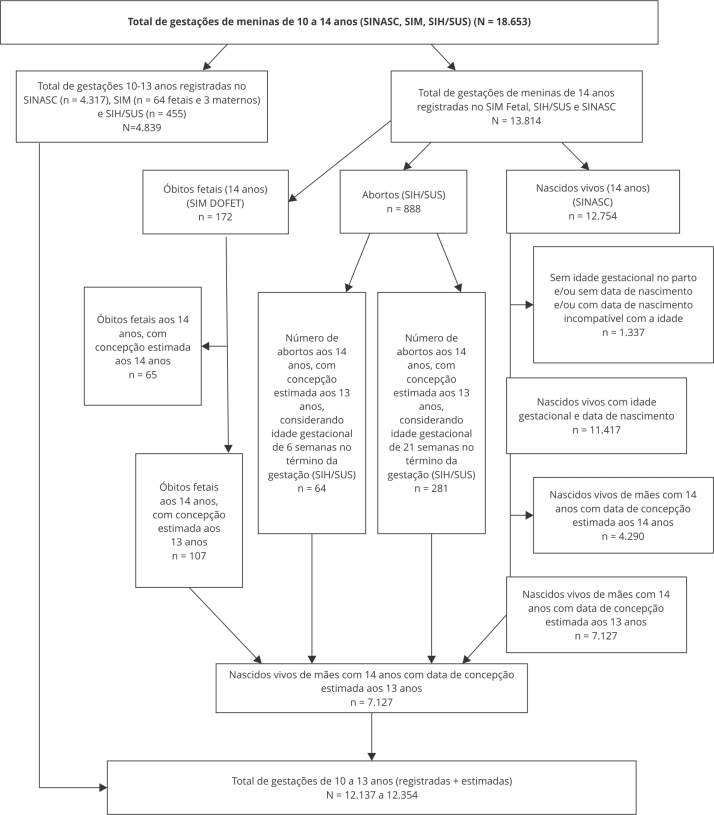
Número estimado de gestações em meninas de 10 a 13 anos, considerando casos registrados nos sistemas nacionais de informação e casos estimados para meninas de 14 anos (com concepção aos 13). Maranhão, Brasil, 2012-2022.

DOFET: Declaração de Óbito Fetal; SIH/SUS: Sistema de Informações Hospitalares do Sistema Único de Saúde; SIM: Sistema de Informações sobre Mortalidade; SINASC: Sistema de Informações sobre Nascidos Vivos. Fonte: elaboração própria.

No período 2012-2022, foram identificados, em meninas de 10 a 13 anos, 4.317 nascidos vivos no SINASC, 64 óbitos fetais, 3 óbitos maternos ocorridos antes do término na gestação no SIM e 455 internações por aborto no SIH/SUS, resultando num total de 4.839 gestações registradas em meninas de 10 a 13 anos no Estado do Maranhão (Figura 1).

Das 12.754 meninas com término da gestação aos 14 anos identificadas no SINASC, 1.337 foram excluídas por ausência de informação sobre idade gestacional, data de nascimento ou idade incompatível com a data de nascimento. Das 11.417 restantes, estimou-se que 4.290 tinham 14 anos e 7.127 tinham 13 anos na data da concepção da gestação atual, o que corresponde a 62,4% das DNV com informação de idade gestacional e data de nascimento disponível. No SIM, foram identificados 172 óbitos fetais em meninas de 14 anos, sendo estimados que 107 (62,4% do total, mesma proporção observada em nascidos vivos) tiveram data de concepção aos 13 anos. Finalmente, no SIH/SUS, foram identificadas 888 internações por aborto em meninas de 14 anos, com estimativa de que entre 64 e 281 tiveram data de concepção aos 13 anos. No total, estimou-se que entre 12.137 e 12.354 gestações ocorreram em meninas de 10 a 13 anos no Estado do Maranhão (Figura 1), o que corresponde a um aumento de 2,5% em relação ao número de gestações registradas.

**Figura 2:**
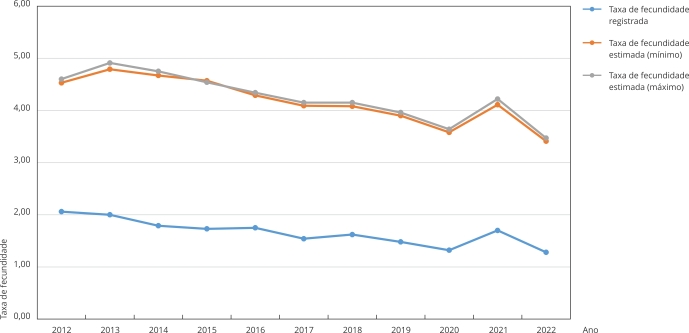
Taxa de fecundidade registrada e estimada de meninas de 10 a 13 anos. Maranhão, Brasil, 2012-2022.

A taxa de fecundidade em meninas de 10 a 13 anos, considerando as gestações registradas, foi de 1,70 por 1.000 no período 2012-2022, com redução de 2,06 por 1.000 em 2012 a 1,28 por 1.000 em 2022, com uma pequena elevação em 2021. Quando consideradas as gestações estimadas, incluindo meninas de 14 anos com data de concepção aos 13 anos, a taxa de fecundidade se eleva para 4,25/4,33 por 1.000 no período, com redução de 4,53/4,60 por 1.000 em 2012 para 3,41/3,47 por 1.000 em 2022, também com pequena elevação em 2021 (Figura 2).

Fonte: Sistema de Informações sobre Mortalidade (SIM), Sistema de Informação sobre Nascidos Vivos (SINASC), Sistema de Informações Hospitalares do Sistema Único de Saúde (SIH/SUS) e Censo Demográfico de 2022 (Instituto Brasileiro de Geografia e Estatística - IBGE).

**Figura 3:**
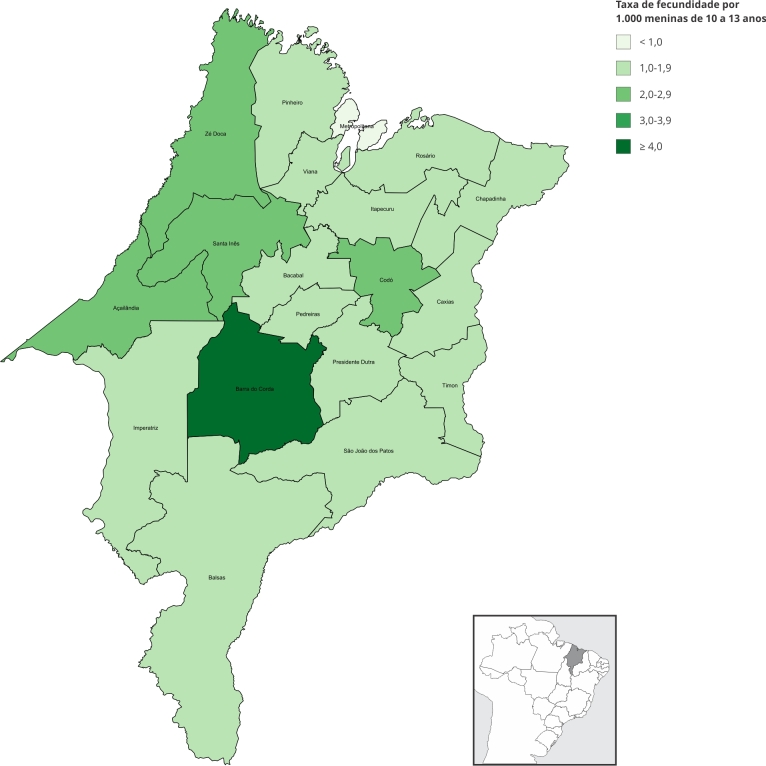
Taxa de fecundidade em meninas de 10 a 13 anos por Unidade Regional de Saúde. Maranhão, Brasil, 2012-2022.

A distribuição do número de gestações e da taxa de fecundidade registrada em meninas de 10 a 13 anos variou nas diferentes URS do estado (Figura 3), sendo mais elevada na URS Barra do Corda (4,22 por 1.000) e mais baixa na URS Metropolitana (0,86 por 1.000).

Fonte: Sistema de Informações sobre Mortalidade (SIM), Sistema de Informação sobre Nascidos Vivos (SINASC), Sistema de Informações Hospitalares do Sistema Único de Saúde (SIH/SUS) e Censo Demográfico de 2022 (Instituto Brasileiro de Geografia e Estatística - IBGE).

**Figura 4:**
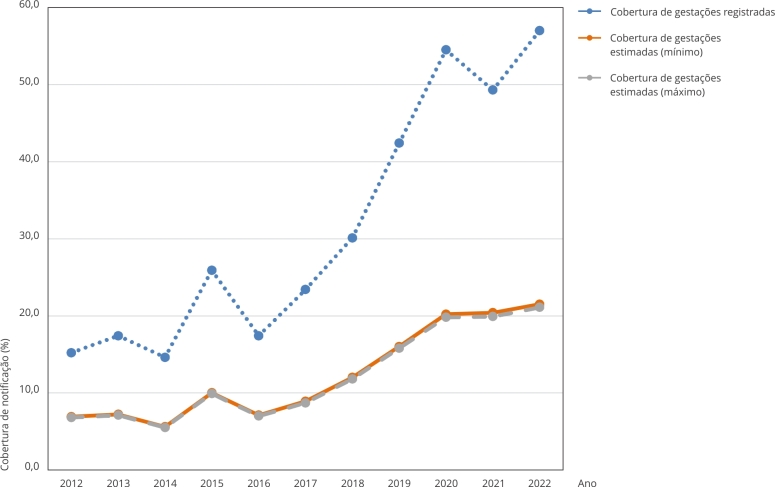
Estimativa da cobertura de notificação de casos de estupro de meninas de 10 a 13 anos. Maranhão, Brasil, 2012-2022.

**Figura 5:**
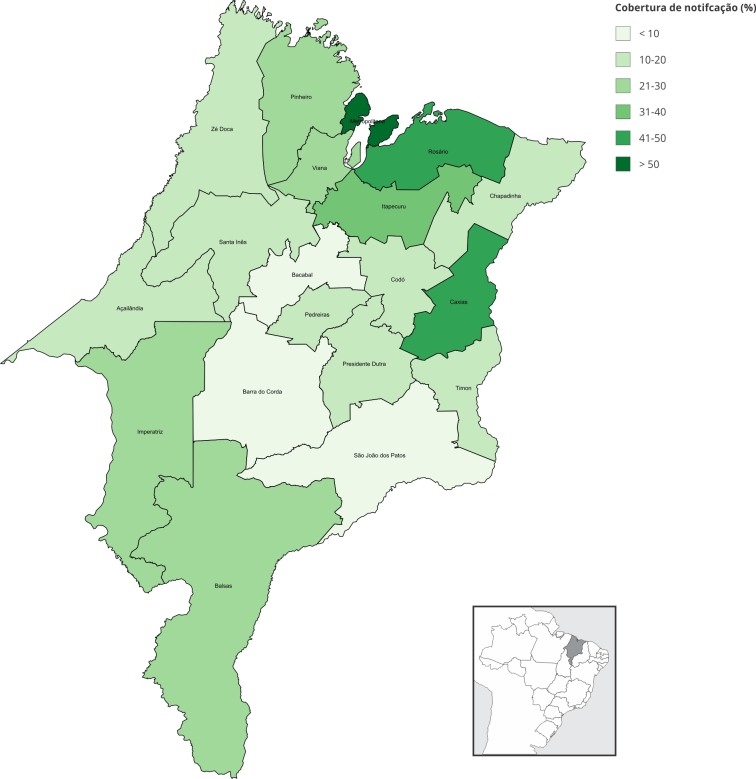
Cobertura de notificação de casos de estupro de meninas de 10 a 13 anos por Unidade Regional de Saúde. Maranhão, Brasil, 2012-2022.

Quanto às notificações de casos de estupro, foram identificados 1.410 casos em meninas de 10 a 13 anos no Maranhão no período de 2012 a 2022, resultando numa cobertura estimada de 29,1% no período ao considerar as gestações registradas. Ao contabilizar as gestações estimadas, a cobertura de notificação se reduz para 11,4%/11,6%. Nota-se um aumento da cobertura de notificação ao longo dos anos, alcançando 57% para casos registrados e 21,5%/21,1% para casos estimados no ano de 2022 (Figura 4). A cobertura de notificação, considerando apenas as gestações registradas, variou segundo as URS no período 2012-2022 (Figura 5), sendo as menores observadas em São João dos Patos (5,9%), Bacabal (6,1%) e Barra do Corda (9,3%), e a maior na URS Metropolitana (126,7%).

Quanto aos desfechos das gestações registradas em meninas de 10 a 13 anos e mulheres de 20 a 29 anos no Estado do Maranhão no período de 2012 a 2022, observa-se valores mais elevados para todos os desfechos em meninas de 10 a 13 anos, exceto a razão aborto legal/casos notificados de estupro, cujo valor foi muito inferior em meninas de 10 a 13 anos (0,8%) quando comparado ao observado em mulheres de 20 a 29 anos (33,9%) (Tabela 1). De um modo geral, os valores encontrados para os desfechos analisados em meninas de 10 a 13 anos foram aproximadamente 2 vezes mais elevados do que em mulheres de 20 a 29 anos, com exceção da RMM, que foi 4 vezes maior, e da taxa de mortalidade fetal, que foi 1,28 maior.

Fonte: Sistema de Informações sobre Mortalidade (SIM), Sistema de Informação sobre Nascidos Vivos (SINASC), Sistema de Informações Hospitalares do Sistema Único de Saúde (SIH/SUS) e Censo Demográfico de 2022 (Instituto Brasileiro de Geografia e Estatística - IBGE).

Fonte: Sistema de Informações sobre Mortalidade (SIM), Sistema de Informação sobre Nascidos Vivos (SINASC), Sistema de Informações Hospitalares do Sistema Único de Saúde (SIH/SUS) e Censo Demográfico de 2022 (Instituto Brasileiro de Geografia e Estatística - IBGE).

**Tabela 1:** Desfechos da gestação de meninas de 10 a 13 anos e mulheres de 20 a 29 anos. Maranhão, Brasil, 2012-2022.

Desfecho da gestação	10-13 anos	20-29 anos	Razão 10-13/20-29
Porcentagem de nascidos vivos prematuros (idade gestacional < 37 semanas)	18,5	9,9	1,86
Porcentagem de nascidos vivos com baixo peso ao nascer (peso < 2.500g)	14,2	6,6	2,16
Taxa de mortalidade fetal por 1.000 nascidos vivos	14,6	11,4	1,28
Taxa de mortalidade neonatal total por 1.000 nascidos vivos	16,4	8,8	1,87
Razão de mortalidade materna por 100.000 nascidos vivos	301,1	69,8	4,32
Razão aborto legal/casos de estupro notificados	0,8	33,9	0,02

## Discussão

Os resultados deste estudo mostram taxa de fecundidade de 1,7 por 1.000 em menores de 14 anos no Maranhão (2012-2022), aumentando 2,5 vezes ao incluir meninas com data provável de concepção aos 13 e com término da gestação aos 14 anos. Houve redução da taxa de fecundidade no período, com pequena elevação em 2021, além de grande variação entre URS. A cobertura de notificação de estupro foi de 1/3 dos casos registrados e 11% dos casos estimados. Todos os desfechos analisados, exceto a razão aborto legal/estupros notificados, foram 1,28 a 4,32 vezes mais frequentes em meninas de 10 a 13 anos do que em mulheres de 20 a 29 anos, sendo que menos de 1% das gestações em meninas de 10 a 13 anos resultaram em aborto legal.

Neste estudo, foi adotada metodologia similar à utilizada por Taquette et al. ^2^, que estimaram o total de gestações em meninas de 10 a 13 anos, incluindo gestações de meninas de 14 anos com provável concepção aos 13 anos. Entretanto, foram adotados procedimentos diferentes para estimar as gestações com provável concepção aos 13 anos, principalmente nas gestações com desfecho óbito fetal, óbito materno e aborto. No estudo de Taquette et al. ^2^, foi utilizada a proporção da população feminina de 10 a 13, dentre o total na faixa de 10 a 14 anos, para estimar óbitos fetais e abortos em meninas de 10 a 13 anos. Já neste estudo, utilizamos estratégias específicas para as bases do SIM fetal e SIH/SUS, além da inclusão de gestações que terminaram em óbito materno antes da expulsão do feto. Essas diferenças metodológicas podem explicar as diferenças observadas nos dois estudos. No estudo de Taquette et al. ^2^, foi identificado um aumento de três vezes no número de gestações ao considerar casos estimados, enquanto neste estudo esse aumento foi de 2,5. Além disso, no estudo de Taquette et al. ^2^, foram feitas estimativas para as macrorregiões do Brasil como unidade de análise, enquanto o presente estudo adota as URS, o que permite uma abordagem mais específica do contexto do Maranhão. Ressalta-se que a abordagem proposta por Taquette et al. ^2^, e aprimorada neste estudo, visa melhorar as estimativas de gestações em meninas de 10 a 13 anos, que são, por definição, resultantes de estupro. Não existe uma base de dados que contenha dados relativos às gestações resultantes de relações sexuais ocorridas em meninas de 10 a 13 anos que permitisse a validação da metodologia proposta. Os cálculos, portanto, são baseados em uma formulação teórica, que considera a duração da gestação para estimar a data provável da concepção e a idade materna nessa ocasião.

A taxa de fecundidade encontrada neste estudo para o período 2012 a 2022, considerando o total de gestações estimadas, foi similar à observada nas regiões Norte e Nordeste e 2 a 3 vezes superior às estimadas para as regiões Sul e Sudeste no período de 2012 a 2018 ^2^. A redução do número de gestações e da taxa de fecundidade no período analisado é compatível com dados de outros estudos que também evidenciaram redução da taxa de fecundidade em adolescentes no país ^2^ ^,^ ^6^ ^,^ ^11^.

Neste estudo, observou-se pequena elevação da taxa de fecundidade no ano de 2021. Uma hipótese para esse aumento é a ocorrência da pandemia de COVID-19, já que existem relatos de crescimento na ocorrência de violência dentro de casa e de abusos sexuais contra mulheres e meninas por conta do confinamento e do maior período de permanência nas residências ^12^ ^,^ ^13^, além de redução no acesso a serviços de saúde sexual e reprodutiva, o que pode ter contribuído para um aumento de gestações não planejadas, maternidades forçadas e abortos realizados em condições de risco ^14^.

A taxa de fecundidade apresentou grande variação entre as URS do estado, heterogeneidade que pode estar associada a fatores locais como pior condição socioeconômica medida pelo baixo Índice de Desenvolvimento Humano Municipal (IDHM), acesso limitado a serviços de saúde sexual e reprodutiva e influências culturais, com diferentes percepções acerca de gestações em meninas de 10 a 13 anos, que podem não ser percebidas como fruto de violência sexual. Outra hipótese seria a maior proporção de indígenas em algumas URS do estado que apresentaram elevada taxa de fecundidade, como a URS Barra do Corda. Jovens indígenas apresentam menor grau de escolaridade, o que é um fator associado a gestações precoces. Além disso, os padrões culturais em algumas comunidades indígenas podem favorecer casamentos ou uniões consensuais precoces. Esses arranjos sociais, muitas vezes associados às tradições locais, levam a uma maior proporção de gestações na adolescência em comparação com outros grupos raciais. Estima-se que o percentual de nascidos vivos entre meninas indígenas de 10 a 14 anos seja quase quatro vezes maior do que entre meninas brancas ^15^.

Observou-se aumento da cobertura de notificação ao longo do período analisado, compatível com estudo que analisou a evolução da notificação de violências praticadas contra adolescentes entre 2015 e 2022, onde foi observado predomínio de notificações no sexo feminino, aumento de notificações na faixa etária entre 10 e 14 anos e aumento do número de notificações de violência sexual ^16^. Ressalta-se que, apesar do aumento, a cobertura de notificação observada neste estudo é baixa, especialmente quando considerados os casos de gestações estimadas. É importante destacar que a cobertura apresentada é superestimada, pois nem todos os casos de estupro resultam em gestações. Ou seja, a cobertura de notificação de todos os casos de estupro é ainda mais baixa, o que afeta a correta avaliação epidemiológica dessa ocorrência.

Observou-se ainda, heterogeneidade na cobertura de notificação entre as URS do estado. Na URS Metropolitana, a cobertura superior a 100% provavelmente reflete a existência de serviços de referência que atendem casos de residentes de outras regiões, sendo esses casos computados na cobertura local. Regiões com elevada taxa de fecundidade apresentaram coberturas mais baixas de notificação, como a URS de Barra do Corda, reforçando a hipótese de que essas gestações precoces não sejam percebidas como casos de violência sexual.

A subnotificação do estupro entre adolescentes pode decorrer da omissão familiar, do medo ou desconhecimento de direitos, do não acesso aos serviços e do despreparo dos profissionais em notificar. Muitos profissionais de saúde desconhecem sua responsabilidade na Rede de Proteção dos Direitos da Criança e do Adolescente, comprometendo a efetividade da proteção integral. A falta de capacitação e de protocolos padronizados pode dificultar o reconhecimento da violência sexual, o atendimento adequado e a articulação intersetorial, limitando o acompanhamento dos casos de violência infantil. Embora conselheiros tutelares reconheçam a saúde como canal essencial de detecção e encaminhamento, na prática, o setor concentra-se no tratamento de lesões físicas, reforçando um modelo biomédico e curativo. Isso impede um cuidado integrado com prevenção, diagnóstico e acompanhamento em parceria com outras áreas, resultando em atendimento fragmentado e na manutenção do ciclo de violência ^17^.

A menor razão entre abortos legais/casos notificados de estupro em meninas de 10 a 13 anos, em comparação a mulheres de 20 a 29, evidencia obstáculos ao direito de interromper gestações resultantes de estupro. Foram considerados apenas os abortos legais frente aos estupros notificados, mas, como toda gestação em menor de 14 anos configura estupro de vulnerável, a proporção de gestações que resultou em aborto legal é ainda menor. Muitas adolescentes desconhecem a possibilidade de aborto legal em casos de estupro, risco de vida materna ou anencefalia fetal, o que reflete falhas na comunicação sobre direitos reprodutivos e serviços disponíveis, sobretudo em contextos de violência sexual ^18^. Além disso, impulsionado por movimentos conservadores, novas tentativas de restrição de acesso ao aborto têm surgido, a exemplo do *Projeto de Lei nº 1.904/2024*, que criminaliza abortos após 22 semanas de gestação ^19^. Essas iniciativas penalizam todas as vítimas de estupro, mas especialmente meninas de 10 a 14 anos, o que coloca em risco sua saúde e direitos ^20^.

Meninas dessa faixa etária apresentam dificuldades para o diagnóstico precoce da gestação e dependem de familiares ou tutores para autorizar o aborto legal. A dinâmica de poder, sobretudo quando o agressor é conhecido, pode atrasar ou impedir essa busca, principalmente em cenários de abuso familiar, onde a proteção legal e o suporte social são menos acessíveis ^18^. O estigma social, que responsabiliza e julga severamente meninas grávidas, dificulta a busca por ajuda e gera vergonha e isolamento. Além disso, profissionais de saúde despreparados podem reforçar preconceitos ao questionar a violência ou exigir documentos desnecessários, influenciados até por crenças religiosas ^18^. A ausência de serviços especializados em áreas rurais e periféricas limita ainda mais o acesso dessas meninas. Em 2021, um estudo nacional mostrou que apenas 55 municípios no Brasil estavam aptos a oferecer Serviços de Referência para Interrupção de Gravidez nos casos previstos em lei, concentrados sobretudo em centros urbanos do Sul e Sudeste, evidenciando o menor acesso nas regiões Norte e Nordeste ^21^. A concentração de unidades hospitalares que realizam abortos legais em grandes centros urbanos, como a URS Metropolitana, reduz a acessibilidade e impõe custos com os quais muitas mulheres não conseguem arcar ^18^. A Organização Mundial da Saúde (OMS) ^22^ alerta que a restrição ao abortamento seguro aumenta riscos à saúde e amplia desigualdades sociais.

A maior frequência de desfechos negativos em gestações de 10 a 13 anos encontrada neste estudo é coerente com outros estudos sobre o tema. Pesquisas anteriores apontam que adolescentes grávidas, bem como mulheres vítimas de estupro, tem maior probabilidade de parto prematuro ^23^. O baixo peso ao nascer, frequentemente relacionado à vulnerabilidade social, também é um desfecho mais comum em recém-nascidos de mães adolescentes. A maior RMM é compatível com dados nacionais, que mostram RMM 2 a 3 vezes mais elevada em gestantes de 10 a 14 anos. Já a taxa de mortalidade fetal, de 14,6 por 1.000 nascidos, e a taxa de mortalidade neonatal, de 16,4 por 1.000 nascido vivo, ambas para o período 2012-2022, também foram superiores às observadas no país no mesmo período, de 9,9 por 1.000 e 8,7 por 1.000, respectivamente, conforme dados do SIM ^24^. A maior frequência desses desfechos negativos pode ser atribuída às gestações de alto risco devido à idade e às condições socioeconômicas precárias, incluindo menor acesso a serviços de pré-natal ^23^, destacando a importância de políticas públicas voltadas à proteção e assistência a essas meninas e à redução desses indicadores ^25^.

Este estudo utilizou bases de dados secundários, estando sujeito a problemas de cobertura e qualidade de registro. A cobertura do SINASC e do SIM no estado é próxima a 100% ^7^, enquanto a cobertura do SIH/SUS para registro de partos é próxima a 80% ^26^. Não foram identificados estudos que tenham avaliado a cobertura de registro de abortos no estado, embora o registro de abortos no SIH/SUS no país apresente boa cobertura ^27^. Portanto, embora problemas de registro possam existir, espera-se um efeito pequeno nas estimativas apresentadas. Quanto à qualidade da informação, a falta de informações completas sobre idade gestacional e data de nascimento da mãe resultou na exclusão de 1.337 DNV. A base de dados do SIH/SUS não inclui informações sobre a data de término da gestação nem sobre a idade gestacional, enquanto o SIM não possui a data de nascimento da mãe. Essas limitações restringiram a possibilidade de estimar com precisão o número de gestações que resultaram em aborto ou óbito materno e fetal ocorridas em meninas de 14 anos cuja concepção ocorreu aos 13 anos. Para óbitos fetais, na ausência de outro parâmetro, consideramos que a mesma proporção observada em nascidos vivos ocorreria nos óbitos fetais. Entretanto, é possível que esse parâmetro seja conservador, dada a maior frequência de desfechos negativos em meninas de 10 a 13 anos. Para os casos de aborto, utilizamos dois parâmetros de idade gestacional no término da gestação, visando gerar estimativas mínimas e máximas para esse desfecho. Também não foram analisadas internações por aborto na saúde privada. Entretanto, como a cobertura de planos de saúde no estado é inferior a 10% no período analisado, espera-se pouco efeito nas estimativas apresentadas.

## Conclusão

A elevada taxa de fecundidade em meninas de 10 a 13 anos no Estado do Maranhão, principalmente ao incluir gestações com término aos 14 anos, a subnotificação de casos de estupro, a elevada frequência de desfechos negativos, a baixa frequência de abortos legais, bem como a heterogeneidade entre as URS, indica desigualdades sociais e limitações nas políticas de saúde sexual e reprodutiva no estado, incluindo o enfrentamento da gravidez precoce e da violência sexual. A gravidez em meninas dessa faixa etária não deve ser compreendida como um evento isolado, mas sim como um marcador de vulnerabilidade e violação de direitos ^23^. A gravidez precoce representa não apenas um impacto significativo para a saúde física, emocional e social das vítimas, mas também um indicativo de falhas estruturais na proteção à infância e na resposta institucional à violência. As consequências dessas condições vão além da saúde, perpetuando ciclos de pobreza e desigualdades que atingem não só as vítimas, mas também suas famílias e comunidades ^3^. A subnotificação dos casos, aliada às barreiras no acesso a serviços essenciais, reforça a necessidade de aprimoramento das estratégias de vigilância e assistência.

## Data Availability

Os dados de pesquisa estão disponíveis mediante solicitação à autora de correspondência.
